# bZIP17 regulates heat stress tolerance at reproductive stage in *Arabidopsis*

**DOI:** 10.1007/s42994-021-00062-1

**Published:** 2021-11-25

**Authors:** Juan Gao, Mei-Jing Wang, Jing-Jing Wang, Hai-Ping Lu, Jian-Xiang Liu

**Affiliations:** State Key Laboratory of Plant Physiology and Biochemistry, College of Life Sciences, Zhejiang University, Hangzhou, 310027 China

**Keywords:** *Arabidopsis*, bZIP17, Flower, Fertility, Heat Stress, UPR

## Abstract

High temperature elicits a well-conserved response called the unfolded protein response (UPR) to bring protein homeostasis in the endoplasmic reticulum (ER). Two key UPR regulators bZIP28 and bZIP60 have been shown to be essential for maintaining fertility under heat stress conditions in *Arabidopsis*, however, the function of transcriptional activator bZIP17, a paralog of bZIP28, in heat stress response at reproductive stage is not reported. Here we found that *bzip17* mutant plants were sensitive to heat stress in terms of silique length and fertility comparing to that of wildtype (WT) *Arabidopsis* plants, and transcriptomic analysis showed that 1380 genes were specifically up-regulated and 493 genes were specifically down-regulated by heat stress in the flowers of WT plants comparing to that in *bzip17* mutant plants. These bZIP17-dependent up-regulated genes were enriched in responses to abiotic stresses such as water deprivation and salt stress. Further chromatin immuno-precipitation coupled with high-throughput sequencing (ChIP-Seq) uncovered 1645 genes that were direct targets of bZIP17 in *MYC-bZIP17* expressing seedlings subjected to heat stress. Among these 1645 genes, ERSE-II *cis*-element was enriched in the binding peaks of their promoters, and the up-regulation of 113 genes by heat stress in flowers was dependent on bZIP17. Our results revealed direct targets of bZIP17 in flowers during heat stress responses and demonstrated the important role of bZIP17 in maintaining fertility upon heat stress in plants.

**Supplementary Information:**

The online version contains supplementary material available at 10.1007/s42994-021-00062-1.

## Introduction

Global warming has great impact on plant growth and food production, as a yield loss of 6–7% per 1 °C increase in seasonal mean weather associated with extreme heat stress predicted (Lesk et al. 2016). Understanding on how plants perceive and respond to high temperature signals, especially at reproductive stage, is vital for molecular breeding of temperature stress resilient crops (Zhang et al. 2019).

A primary effect of heat stress on cellular function in plants is denaturing proteins and causing protein aggregations both in the cytosol and endoplasmic reticulum (ER) (Ding et al. 2020). Therefore, intracellular protein homeostasis is important to maintain the protein conformation, status, level and activity during heat stress (Sun et al. 2021). Indeed, a major thermotolerance quantitative trait loci (QTL) from African rice (*Oryza glaberrima*), Thermotolerance 1 (*OgTT1*), was identified, and it encodes an α2 subunit of 26S proteasome responsible for eliminating cytotoxic denatured proteins (Li et al. 2015). A number of heat shock proteins (HSPs), such as HSP100s, HSP90s, HSP70s, HSP60s, HSP40s and sHSPs with different molecular weight, have been characterized with the major functions of these proteins as molecular chaperons in cytoplasm during heat stress responses (Jacob et al. 2017; Nover and Scharf 1997). In addition, heat stress transcription factors (HSFs) play a crucial role in heat stress responses through regulating the expression of HSPs, providing heat stress tolerance in plants (Guo et al. 2016).

Disturbance of protein folding homeostasis in ER under heat stress conditions elicits a well-conserved unfolded protein response (UPR), in which several membrane-associated transcription factors sense and transduce the stress signals to nucleus to regulate downstream genes, bringing back the protein homeostasis (Liu and Howell 2016). Upon the accumulation of misfolded proteins in the ER, the *Arabidopsis* ER membrane-associated transcription factor AtbZIP28 is translocated from ER to Golgi where it is subjected to intramembrane proteolysis by Golgi-localized proteases (Che et al. 2010; Gao et al. 2008; Liu et al. 2007a). The activated AtbZIP28 enters nucleus and regulates downstream genes to enhance protein folding capacity and accelerate protein trafficking and ER-associated protein degradation (ERAD) (Liu and Howell 2010a; Tajima et al. 2008). However, activation of AtbZIP60 is dependent on the ER-localized AtIRE1 proteins in *Arabidopsis* (Deng et al. 2011; Moreno et al. 2012; Nagashima et al. 2011). Accumulation of misfolded proteins in ER triggers the activation of AtIRE1s, which unconventionally splices *AtbZIP60* mRNA in the double stem-loops at the cytoplasmic side, causing an open reading frame (ORF) shift and elimination of the transmembrane domain in AtbZIP60 (Deng et al. 2011, 2016). In the nucleus, AtbZIP60 interacts with AtbZIP28 and regulate downstream genes alone or together (Iwata and Koizumi 2005; Song et al. 2015; Tajima et al. 2008). *OsNTL3*, a homolog of *AtNAC062*, is up-regulated by OsbZIP74 (ortholog of AtbZIP60) under heat stress conditions and encodes a plasma membrane-tethered membrane associated transcription factor in rice (Hayashi et al. 2012; Liu et al. 2020; Lu et al. 2012; Yang et al. 2014). It is activated and relocates to the nucleus to up-regulate downstream genes involved in UPR and reactive oxygen species (ROS) scavenging, which is essential for heat stress tolerance in rice (Liu et al. 2020).

Relative to vegetative growth, reproductive development is more sensitive to environmental stresses such as heat stress (Barnabas et al. 2008; Begcy et al. 2019; Chaturvedi et al. 2021). In *Arabidopsis*, the *AtbZIP60* promoter is highly active in flowers especially in microspores and tapetum cells, and the *bZIP60* mRNA is constitutively spliced/activated in anthers under normal growth conditions suggesting its role in reproductive development (Deng et al. 2016; Iwata et al. 2008). When stressed at reproductive stage, the *bzip28 bzip60* double mutant plants were more sensitive to heat stress with short siliques and reduced fertility comparing to that in wild-type plants, which agrees with the important role of these two transcription factors in heat stress responses in reproductive tissues (Zhang et al. 2017).

Previous researches have demonstrated that AtbZIP17, a paralog of AtbZIP28, is activated in a manner similar to AtbZIP28 under salt stress conditions to desensitize the ABA signaling pathway (Liu et al. 2007b, 2008; Zhou et al. 2015). A recent study showed that AtbZIP17 is required for vegetative development together with AtbZIP28 to mediate the expression of multiple genes involved in cell growth (Kim et al. 2018). Although ER stress and heat stress induce the activation of AtbZIP17 (Che et al. 2010), apparently AtbZIP28 and AtbZIP60 are two major transcription factors for canonical UPR gene expression (Kim et al. 2018; Song et al. 2015), the role of AtbZIP17 in heat stress response is not reported. In the current paper, we found that AtbZIP17 also plays important roles in maintaining fertility under heat stress conditions. Through RNA-seq and ChIP-seq analysis, we further revealed that AtbZIP17 directly binds to 113 genes whose expression is dependent on AtbZIP17 in flowers under heat stress conditions.

## Results

### AtbZIP17 is essential for maintaining fertility under heat stress conditions

**Fig. 1:**
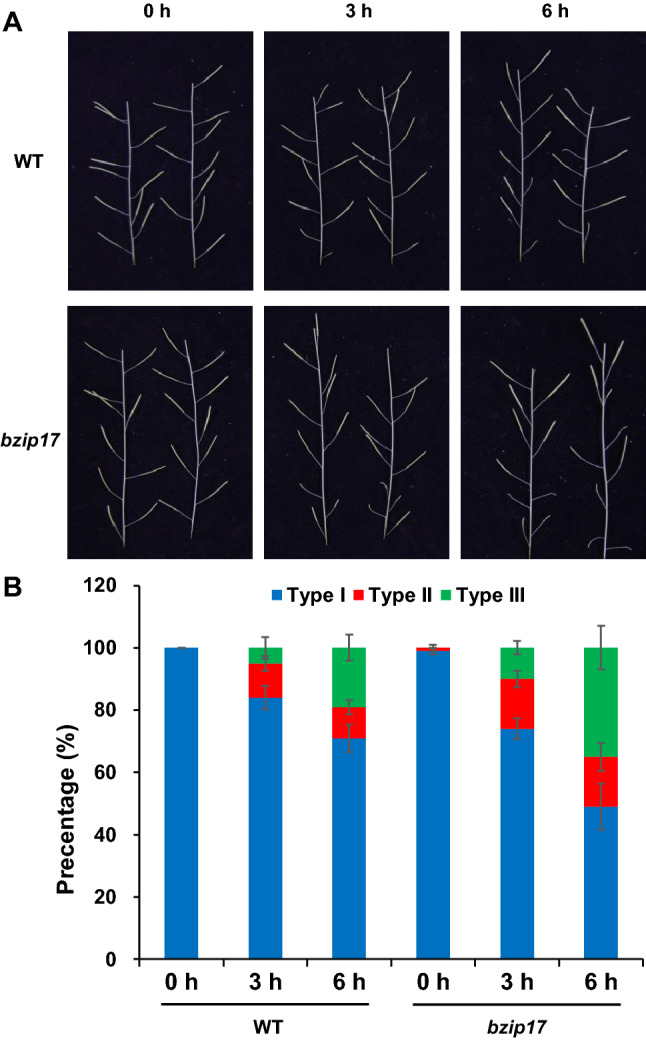
AtbZIP17 is required for maintaining fertility under heat stress conditions. **A** Siliques of *Arabidopsis* wild-type plants (WT) and *AtbZIP17* mutant (*bzip17*) plants at recovery stage after treating with heat stress for different time at the stage of late bolting. **B** Heat stress sensitivity of WT and *bzip17* plants in terms of silique length. Silique lengths were measured and percentage of each type of silique was calculated. Type I, full fertile (> 10 mm in length); Type II, partial sterile (5–10 mm in length); Type III, complete sterile (< 5 mm in length). Bars depict SE (*n* = 10)

Our previous transcriptomic data have shown that *AtbZIP17* is up-regulated by heat stress in *Arabidopsis* flower tissues (Zhang et al. 2017). To examine the role of *AtbZIP17* in maintaining fertility under heat stress conditions, we compared the heat sensitivity of WT and *bzip17* knock-out mutant plants (Liu et al. 2007b) at reproductive stage. Compared to the WT plants, the percentage of type II and type III siliques (reduced fertility) was largely increased in *bzip17* mutant plants after exposure to a short period of heat stress (38 °C) at flowering stage (Fig. 1A, B). These results demonstrated that *AtbZIP17* is essential for thermotolerance at the reproductive stage in *Arabidopsis*.

### AtbZIP17 regulates heat stress responsive genes in flowers

**Fig. 2:**
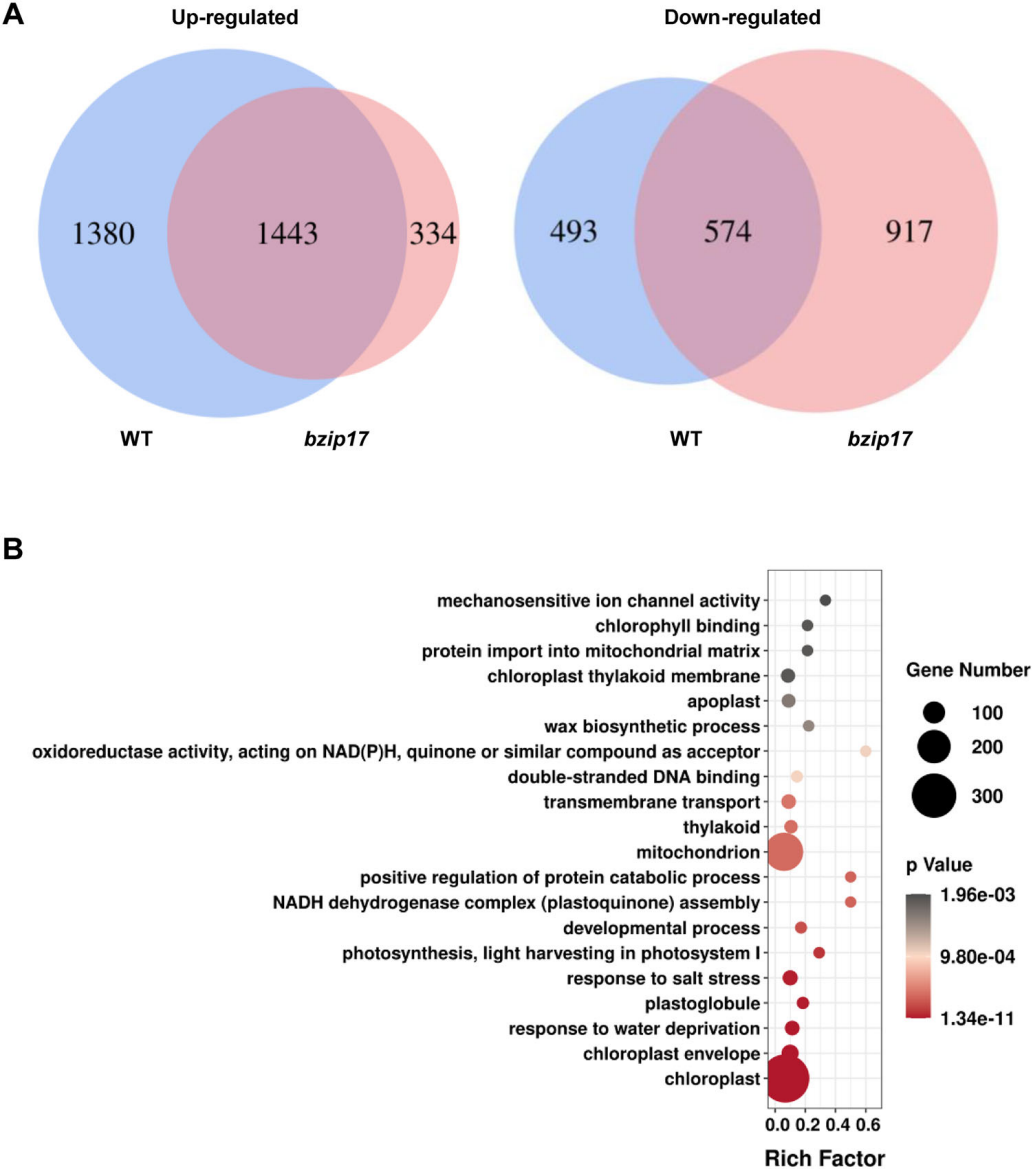
AtbZIP17 regulates downstream genes in flowers under heat stress conditions. **A** Venn diagrams showing the common and specific heat responsiveness in flowers of wild-type plants (WT) and *AtbZIP17* mutant (*bzip17*) plants. **B** GO analysis of AtbZIP17-dependent heat responsive genes in flowers

To understand the function of AtbZIP17 in heat stress responses at reproductive stage, we performed RNA-seq analysis of heat-stressed flowers (stage 1–12) in WT and *bzip17* mutant plants (Supplemental Fig. S1), and found that 1380 and 493 genes were specifically up-regulated [Log_2_(FC) > 2 and *P* < 0.05] and down-regulated [Log_2_(FC) < 0.5 and *P* < 0.05] in WT flowers, respectively (Fig. 2A). There were 334 and 917 genes that were specifically up-regulated and down-regulated in the flowers of *bzip17* mutant plants, respectively (Fig. 2A). Since the activated form of AtbZIP17 has transcriptional activation activity (Liu et al. 2007b), we considered these 1380 genes as AtbZIP17-dependent heat stress responsive genes in flowers (Supplemental Table S1). GO analysis of these genes revealed that ‘response to salt stress’ and ‘response to water deprivation’ were enriched (Fig. 2B). At least eight terms related to chloroplast and photosynthesis were also enriched (Fig. 2B), probably because the green tissue sepal was included in the flower samples. These results supported that AtbZIP17 regulates a number of genes involved in stress responses and chloroplast function in *Arabidopsis* flowers under heat stress conditions.

### AtbZIP17 directly binds to the promoters of stress responsive and UPR genes in seedlings

**Fig. 3:**
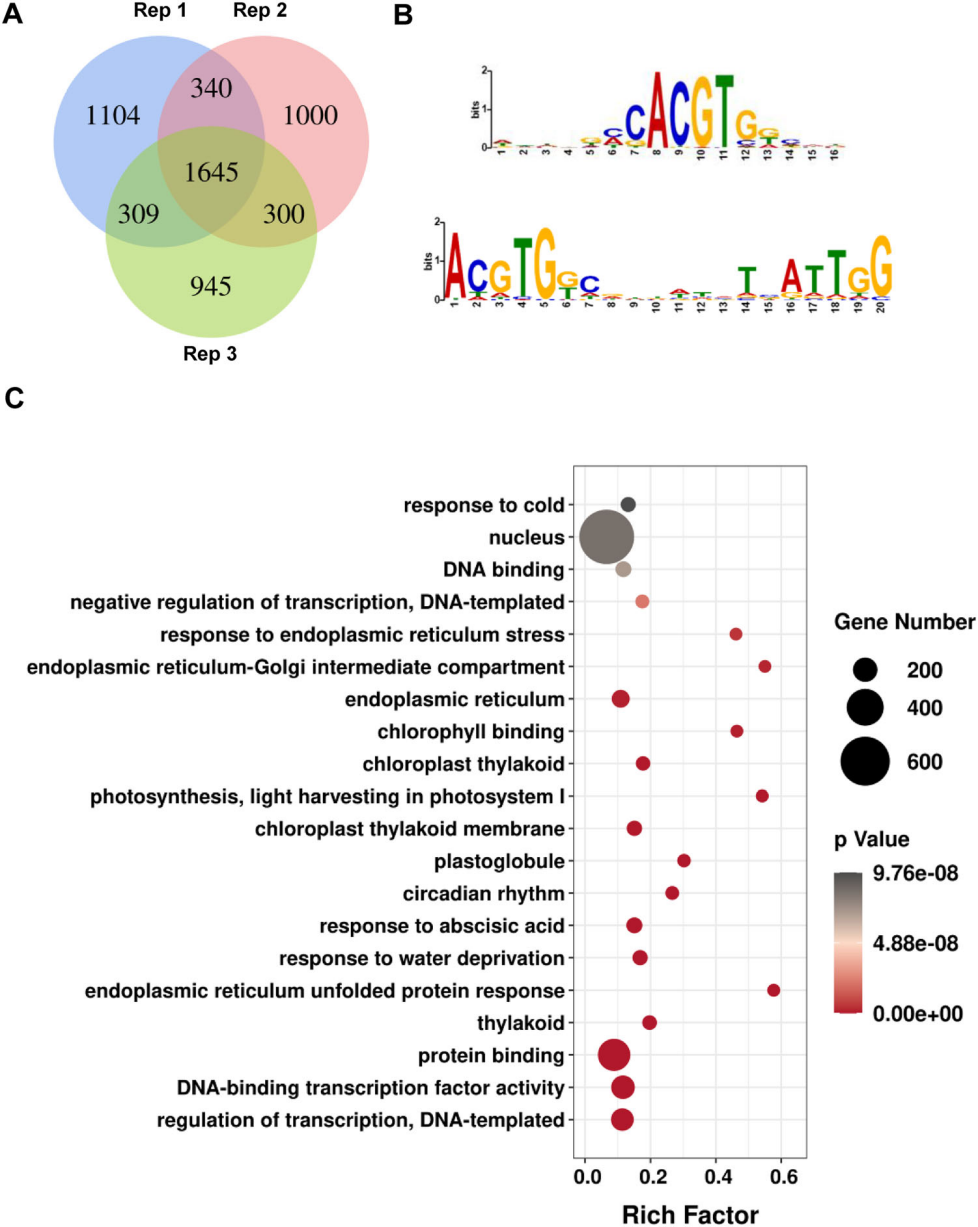
AtbZIP17 directly binds to the promoter of target genes in heat-stressed seedlings. **A** Venn diagram showing the direct targets of AtbZIP17 in three replicates. **B** AtbZIP17-binding motifs in the binding peaks of AtbZIP17 targets. **C** GO analysis of AtbZIP17 direct targets

To reveal genome-wide direct targets of AtbZIP17, we carried out ChIP-seq experiments with heat-stressed *MYC-bZIP17* expressing seedling plants. Western blot analysis confirmed that AtbZIP17 is processed in response to heat stress as the small molecular weight band increased under heat stress conditions (Supplemental Fig. S2). The ChIP-seq results showed that MYC-bZIP17 was enriched in the 2 K upstream sequences of totally 1645 genes in 3 replicates (Fig. 3A and Supplemental Table S2). We considered these genes as the AtbZIP17 direct targets in *Arabidopsis*. For the 133 identified direct targets of AtbZIP28 (Zhang et al. 2017), except AT2G07741, the rest 132 genes are also direct targets of AtbZIP17 as revealed in the current study.

Among the 2629 binding peaks on the promoter regions of these 1645 genes, the bZIP-bind G box and ERSE-II *cis*-elements were significantly enriched (Fig. 3B), which is similar to the binding characteristics of AtbZIP28 (Liu and Howell 2010b; Zhang et al. 2017). Among the proteins encoded by these target genes, ‘response to ABA’, ‘response to water deprivation’, ‘endoplasmic reticulum unfolded protein response’, and subcellular compartments related to the secretory pathway were all significantly enriched (Fig. 3C). These results suggest that AtbZIP17 has both specific and overlapping direct targets with AtbZIP28.

### The expression of AtbZIP17 targets is impaired in flowers of *bzip17* mutant plants under heat stress conditions

**Fig. 4:**
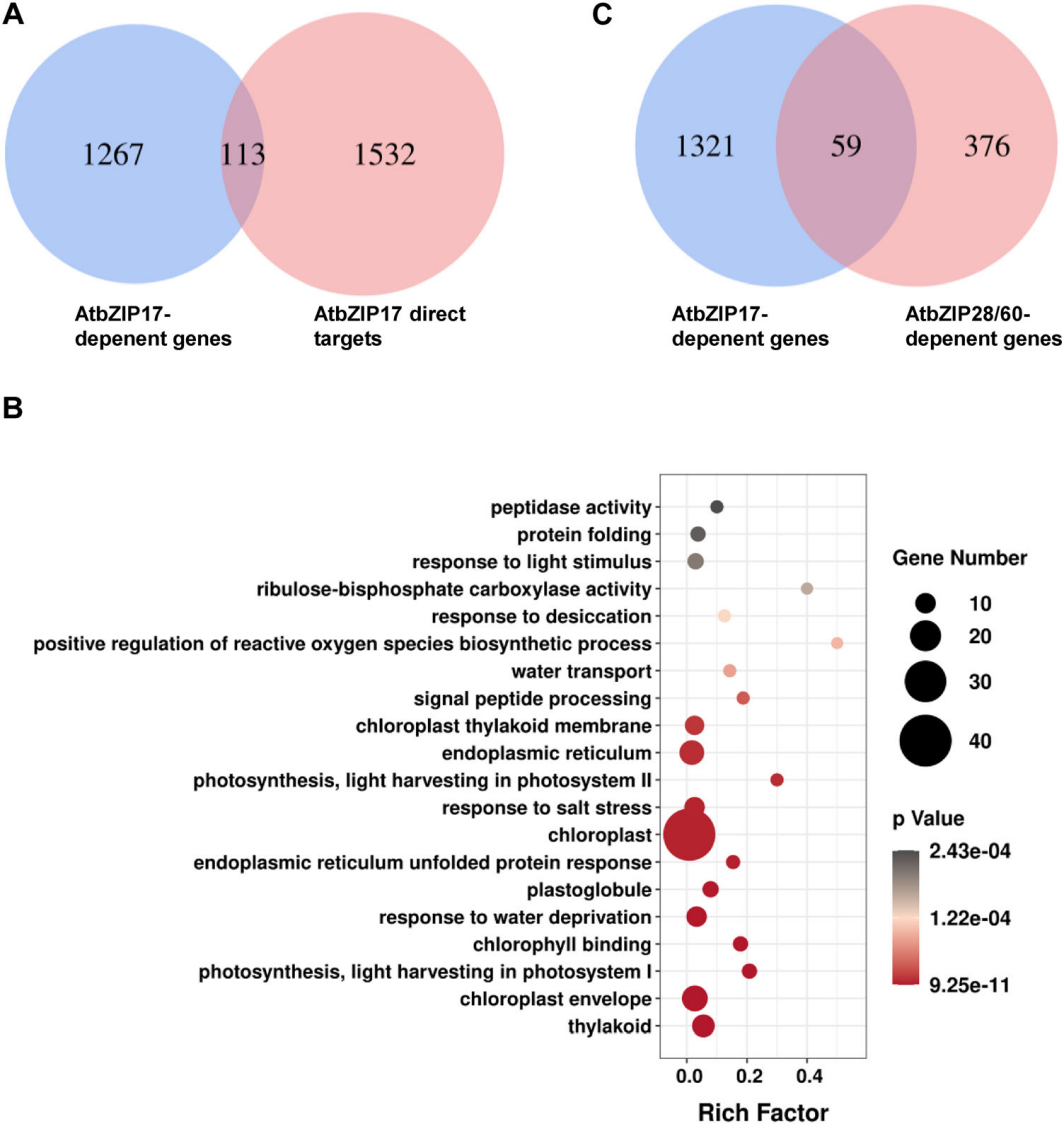
The expression of AtbZIP17 targets is affected in *bzip17* mutant flowers under heat stress conditions. **A** Venn diagram showing 113 direct candidate targets of AtbZIP17 in flowers under heat stress conditions. **B** GO analysis of heat responsive AtbZIP17 direct targets in flowers. **C** Comparison of AtbZIP17-dependent heat responsive genes in flowers and AtbZIP28/60-dependent heat responsive genes in flowers

To know the direct targets of AtbZIP17 in flowers under heat stress conditions, we compared these bZIP17 direct targets in seedlings with those AtbZIP17-dependent heat stress responsive genes in flowers. The results showed that totally 113 target genes were up-regulated by heat at reproductive stage in WT plants but not in *bzip17* mutant plants (Fig. 4A), suggesting that these genes are direct targets of bZIP17 during heat stress response in flowers. GO analysis showed that ‘response to salt stress’, ‘response to water deprivation’, ‘endoplasmic reticulum UPR’, ‘ER’ and eight terms related to chloroplast and photosynthesis were enriched (Fig. 4B). The dominant role of AtbZIP28/60 in transcriptional control of canonical ER stress gene expression (Song et al. 2015) was confirmed in *Arabidopsis* seedlings (Supplemental Fig. S3). We also compared these AtbZIP17-dependent heat stress responsive flower genes to those AtbZIP28/60-dependent heat stress responsive flower genes identified in previous study (Zhang et al. 2017), and found that only 59 genes (4.3%) were also AtbZIP28/60-dependent. Nevertheless, these results revealed direct candidate target genes of AtbZIP17 in flowers and showed that AtbZIP17 has a distinct set of downstream genes to AtbZIP28/60 during heat stress response.

**Fig. 5:**
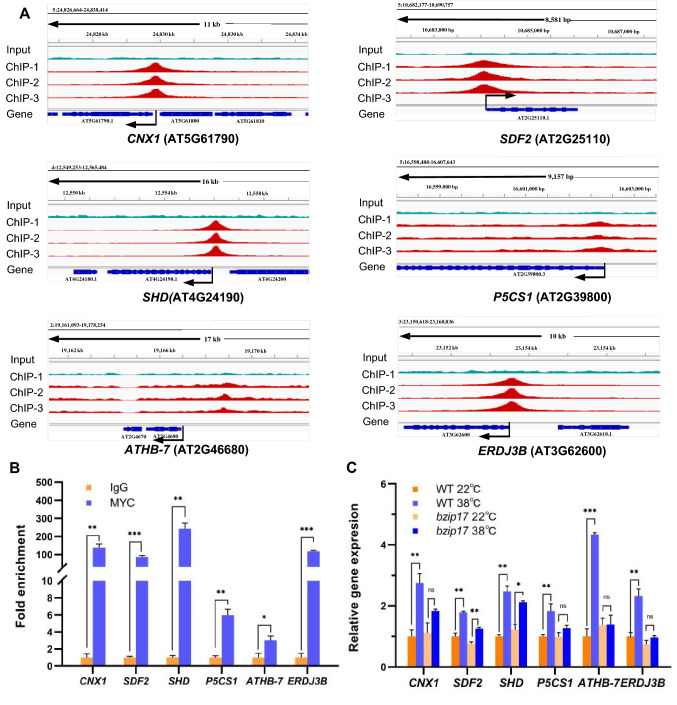
Mutation of *AtbZIP17* impairs the up-regulation of AtbZIP17 direct targets in flowers under heat stress conditions. **A** Distribution of AtbZIP17 binding peaks on six representative target genes in the Integrated Genome Browser. Aligned reads are indicated in red (heat-treated, three replicates) and cyan (input). **B** ChIP-qPCR analysis. Transgenic seedlings overexpressing *MYC-bZIP17* were heat-stressed and harvested for ChIP-qPCR using *anti*-MYC antibody. IgG was used as a negative control. Relative enrichment of each sample was normalized to that of IgG sample, both of which were normalized to that of the *TA3* control. **C** RT-qPCR analysis of AtbZIP17 target genes in flowers under heat stress conditions. Relative gene expression is the expression level of each gene normalized to that in the WT plants, both of which were normalized to that of *PP2A*. The bars depict the *SE* (*n* = 3). ***(*P* < 0.001); **(*P* < 0.01); *(*P* < 0.05); ns, (not significant at *P* < 0.05) in *t* tests

We selected six AtbZIP17 target genes from the ChIP-seq analysis, including four canonical UPR genes (*CNX1*, *SDF2*, *SHD*, and *ERDJ3B*) and two salt stress-related genes (*ATHB-7* and *P5CS1*) whose promoter sequences were enriched by AtbZIP17 (Fig. 5A), and conducted ChIP-qPCR. The results indicated that AtbZIP17 indeed binds to the promoter region of all these six genes (Fig. 5B). To check whether the expression of these target genes was affected by *AtbZIP17* mutation, RT-qPCR was performed. The results showed that all these six genes were significantly up-regulated by heat in WT flowers (Fig. 5C). In contrast, the expression of *CNX1*, *P5CS1*, *ATHB-7*, *ERDJ3B* was not significantly affected by heat stress in *bzip17* mutant flowers (Fig. 5C). Although the expression of *SDF2* and *SHD* was also significantly up-regulated by heat stress in *bzip17* mutant flowers, the fold increase in *bzip17* mutant flowers was lower than that in WT flowers (Fig. 5C). Except *SDF2*, the up-regulation of other five genes by heat stress was not much affected in the *bzip28 bzip60* double mutant plants (Zhang et al. 2017). These results confirmed that AtbZIP17 directly regulates these canonical and noncanonical genes in *Arabidopsis* flowers.

## Discussion

Plant is sensitive to environmental stresses such as drought, salt and heat stresses at reproductive stage, which often results in yield reduction (Barnabas et al. 2008; Begcy et al. 2019; Liu et al. 2006; Ma et al. 2020). Previous results showed that the silique length is corelated to the number of seeds in *Arabidopsis*, using this trait, several QTLs associated with thermotolerance at 35 °C at reproductive stage were identified (Bac-Molenaar et al. 2015), however, the underlying molecular mechanisms are still largely unknown. Through genome-wide association study (GWAS), a cluster of five Immune-associated nucleotide-binding protein (IAN) genes (*IAN2* to *IAN6*) was identified to be responsible for the variation in heat tolerance at reproductive stage in *Arabidopsis* (Lu et al. 2021). The IAN proteins were found to promote cell death induced by heat stress, ER stress, and cell death-inducing molecules (Lu et al. 2021). The association of ER stress response with male reproductive development is widely recognized (Deng et al. 2016; Fragkostefanakis et al. 2016) and several mutants defective in proteins related to protein quality control in plants have pollen development phenotype at normal growth temperature (Singh et al. 2021; Sun et al. 2021). The ER stress response is also essential for thermotolerance at reproductive stage (Singh et al. 2021), and knock-out mutations of *AtbZIP28* and *AtbZIP60*, two major UPR regulators in *Arabidopsis*, confer heat sensitivity at reproductive stage (Zhang et al. 2017). In the current paper, we showed that AtbZIP17 is also important for thermotolerance at reproductive stage, these findings advance our understanding on maintaining fertility under heat stress conditions in plants.

Although AtbZIP17 is activated following treatment with ER stress inducing reagent (Che et al. 2010), the function of AtbZIP17 in canonical ER stress is still an open question. The up-regulation of canonical ER stress genes such as *BiP1*/*2*/*3* and *CRT1* by ER stress was completely blocked in *bzip28 bzip60* double mutant plants (Song et al. 2015; Sun et al. 2013), and the truncated form of AtbZIP17 did not while the truncated form of AtbZIP28 did activate the promoter of *BiP3* in the transient expression assay in protoplasts (Tajima et al. 2008). These results support that AtbZIP17 is not involved in canonical UPR. However, AtbZIP17 has overlapping direct targets with AtbZIP28 as identified in the current study, many of which are known to be important for UPR in plants. Thus, it is possible that AtbZIP17 is still involved in canonical UPR in seedlings, but that function is dependent on the function of AtbZIP28/60, which awaits further investigation in future. The expression of *AtbZIP17*/*28* is not while that of *AtbZIP60* is up-regulated by canonical ER stress in *Arabidopsis* seedlings (Tajima et al. 2008), while the expression of all the three genes *AtbZIP17*/*28*/*60* is up-regulated by heat stress in *Arabidopsis* flowers (Zhang et al. 2017), suggesting that AtbZIP17 is functional redundant to AtbZIP28/60. This is well agreed to our results in the current study that ‘endoplasmic reticulum UPR’ is enriched in the AtbZIP17 direct targets in flowers during heat stress responses. These findings are important for understanding the role of UPR in heat stress response at reproductive stage in plants.

AtbZIP17 regulates vegetative development at normal growth temperature conditions by regulating a different branch of downstream genes to AtbZIP28/60 (Bao et al. 2019; Kim et al. 2018). Several genes such as *ATHB-7*, *LTP3*, and *PP2C*-like were previously identified as the downstream genes of AtbZIP17 under salt stress condition (Henriquez-Valencia et al. 2015; Liu et al. 2007b, 2008). Excessive salt treatment may affect the function and/or constituent of ER, therefore, triggering ER stress response and activation of the ER-associated transcription factor AtbZIP17. However, such ER stress response could be non-canonical UPR (Howell 2017). Indeed, AtbZIP17 is activated by AtS2P following ABA treatment through regulating *ATHB-7* to desensitize the ABA signaling (Zhou et al. 2015). *ATHB-7* is a direct target of AtbZIP17, and it is up-regulated by heat stress in flowers which is dependent on *AtbZIP17*, therefore, AtbZIP17 is involved in ABA signaling during heat stress responses in reproductive tissues in plants.

The GO terms related to chloroplast were enriched in the AtbZIP17 direct targets in flowers during heat stress responses, probably because green sepals were included in the flower samples. Chloroplasts, the photosynthetic organelles of plants, are highly sensitive to heat stress (Hu et al. 2020; Sun et al. 2020). On the other hand, chloroplasts are major resources for ROS production and play an important role in retrograde signaling to protect the integrity and function of chloroplast (Kim 2020; Sun and Guo 2016). Our current results suggest that AtbZIP17 is also important for chloroplast development/function during heat stress response in plants.

## Materials and methods

### Plant material and growing conditions

All plants in this study are in Columbia-0 (Col-0) background. The T-DNA insertional mutant *bzip17* (SALK_104326) was obtained from the Arabidopsis Biological Resource Center (ABRC) (Liu et al. 2007b). The seeds were washed with 0.01% sodium hypochlorite solution for 20 min, and then washed five times with sterile water in an ultra-clean bench. They were grown directly on half-strength Murashige and Skoog (MS) medium (with 1.2% sucrose and 0.7% agar, pH 5.7). After 3 days of vernalization at 4 °C, they were transferred to a standard plant incubator (22 °C, 16/8-h day/night and 60% humidity). For phenotypic analysis and RNA-seq analysis, the seedlings were grown in an incubator for 10 days and then transplanted into a commercial soilless (mixed which include peat, vermiculite, and perlite) under the same conditions.

### Phenotype analysis

Phenotypic analysis of heat stress sensitivity during reproductive stage was performed as followings. After grown at 22 °C until flowering, the unopened floral buds (approximately stage 12) were marked with colored threads/marks, and then placed in the incubator at 38 °C for 3 h and 6 h, respectively. The control plants were grown at 22 °C with the same condition. After heat stress treatment, the plants were transferred to 22 °C and grew further for 10 days. The main inflorescence starting from the mark was selected, and the length of 10 siliques from the mark was measured in ten biological replicates. The siliques were divided into three categories: fully fertile (type I, length > 10 mm), partially sterile (type II, length 5–10 mm) and completely sterile (type III, length < 5 mm) according to the protocols in our previous study (Zhang et al. 2017).

### RNA-seq and RT-qPCR analysis

For RNA-seq analysis, wild-type and *bzip17* mutant plants at flowering stage were treated at 38 °C for 6 h, and the control plants were kept at 22 °C. The unopened floral buds (stage 1–12) from eight plants were collected and immediately frozen in liquid nitrogen with a total of three biological replicates for each treatment. Total RNA was extracted with Trizol (Invitrogen, Shanghai, USA). The cDNA library was constructed according to standard protocols on Illumina Novaseq™ 6000 (LC-Bio Technology Co., Ltd., Hangzhou, China). Cutadapt software is used to remove reads that contain adapter contamination, and to remove low-quality bases and undetermined bases. The HISAT2 software was used to map the reads to the genome and assembled by StringTie with default parameters. Then, all transcriptomes from all samples were merged to reconstruct a comprehensive transcriptome using gffcompare software. After the final transcriptome was generated, StringTie and ballgown were used to estimate the expression levels of all transcripts by calculating FPKM (fragments per kilo-base of transcript per million of mapped reads). The differentially expressed mRNAs were selected with fold change > 2 or fold change < 0.5 and *P* value < 0.05 by R package edgeR or DESeq2, and then gene ontology (GO) enrichment analysis was conducted. For RT-qPCR, total RNAs were extracted with RNA Prep Pure Plant kits (Tiangen, Beijing, China) and reverse transcribed using 5 × PrimeScript RT Master Mix (Takara, Dalian, China). RT-qPCR was performed with SuperReal PreMix Color kits (Tiangen, Beijing, China) in a CFX96 real-time system (Bio-Rad, USA). Gene expression level was calculated based on the ΔΔCt (threshold cycle) method. All the primers are included in Supplemental Table S3.

### ChIP-seq assay

For Chromatin immuno-precipitation coupled with high-throughput sequencing (ChIP-seq), 13-day-old 35S_pro_::MYC-bZIP17 seedlings grown at 22 °C were placed at 42 °C for 2 h, and the seedlings were immediately placed in a 1% (v/v) formaldehyde solution [0.4 M sucrose, 10 mM Tris–HCl (pH 8.0), 1 mM EDTA (pH 8.0) 1% formaldehyde and PMSF] under vacuum for fixing the protein and DNA, and 0.125 M glycine was added to terminate the reaction. Samples were washed with sterile water and grinded into powder in liquid nitrogen for ChIP-seq according to previous protocols (Zhang et al. 2017). Briefly, the powder was mixed with the lysis buffer [50 mM HEPES (pH 7.5), 150 mM NaCl, 1 mM EDTA, 1% Triton X-100, 5 mM β-mercaptoethanol, 10% Glycerol and proteinase inhibitor cocktail (Roche, USA)], and then sonicated to obtain almost 200–500 bp chromatin fragment. Protein A-agarose beads (Millipore) and an anti-MYC antibody (Sigma-Aldrich, USA) were used to precipitate the chromatin. The ChIP-DNA and INPUT DNA libraries were constructed and sequenced by Genergy Bio (Shanghai, China) with Illumina Novaseq™ 6000. Skewer software was used to remove low-quality and linker sequence fragments, and to filter sequences less than 50 bp. Bowtie was used to perform unique mapping analysis between the filtered sequencing data and the reference genome to further remove duplicated reads. MACS software was used to do the peak calling. In each replicate, compared with the input, there were higher binding peak intensity and *P* values < 0.001, which was considered as the potential binding sites of AtbZIP17. The related genes where the common binding peak region in the upstream 2 K in three replicates were considered as the potential targets of AtbZIP17. The common binding *cis*-elements were identified using MEME and GO analysis was performed using the R package edgeR. For ChIP-qPCR, 13-day-old seedlings of 35S_pro_::MYC-bZIP17 plants were treated or nontreated with high temperature at 42 °C for 2 h and sampled for ChIP-qPCR. Primers were listed in Supplemental Table S3.

### Western blot analysis

To verify heat-induced activation of MYC-bZIP17, 13-day-old seedlings of 35S_pro_::MYC-bZIP17 plants grown at 22 °C were placed in an incubator at 42 °C for 2 h, and the plants at 22 °C were used as the controls. Samples were extracted with SDS protein extraction buffer [125 mM Tris–HCl (pH 8.0), 375 mM NaCl, 2.5 mM EDTA, 1% SDS and 1% β-mercaptoethanol] and separated on 4–20% (w/v) SDS-PAGE gels. The proteins were transferred with nitrocellulose membranes and MYC-bZIP17 was detected using *anti*-MYC antibody (Sigma-Aldrich, USA) or *anti*-Tubulin (Sigma-Aldrich, USA) with Tanon-5200 Chemiluminescence Imaging System (Tanon, Shanghai, China).

### Accession numbers

RNA-Seq and ChIP-Seq data from this article can be found in Gene Expression Omnibus (GEO) under the accession number (GSE184984).

## Supplementary Information

Below is the link to the electronic supplementary material.

Supplementary file1 (PDF 227 KB)

Supplementary file2 (XLSX 107 KB) Supplemental Table S1 AtbZIP17-dependent heat stress responsive genes in flowers in Arabidopsis

Supplementary file3 (XLSX 632 KB) Supplemental Table S2 AtbZIP17 direct targets in heat-stressed tissues in Arabidopsis

Supplementary file4 (XLSX 12 KB) Supplemental Table S3 Primers used in the study
