# Supplementary material for: bZIP17 regulates heat stress tolerance at reproductive stage in Arabidopsis
Source: aBIOTECH. 2021 Nov 25;3(1):1–11. doi: 10.1007/s42994-021-00062-1 (PMC9590554; doi:10.1007/s42994-021-00062-1)
Supplement: Supplementary file 1 — Supplementary file1 (PDF 227 KB) [file 42994_2021_62_MOESM1_ESM.pdf]

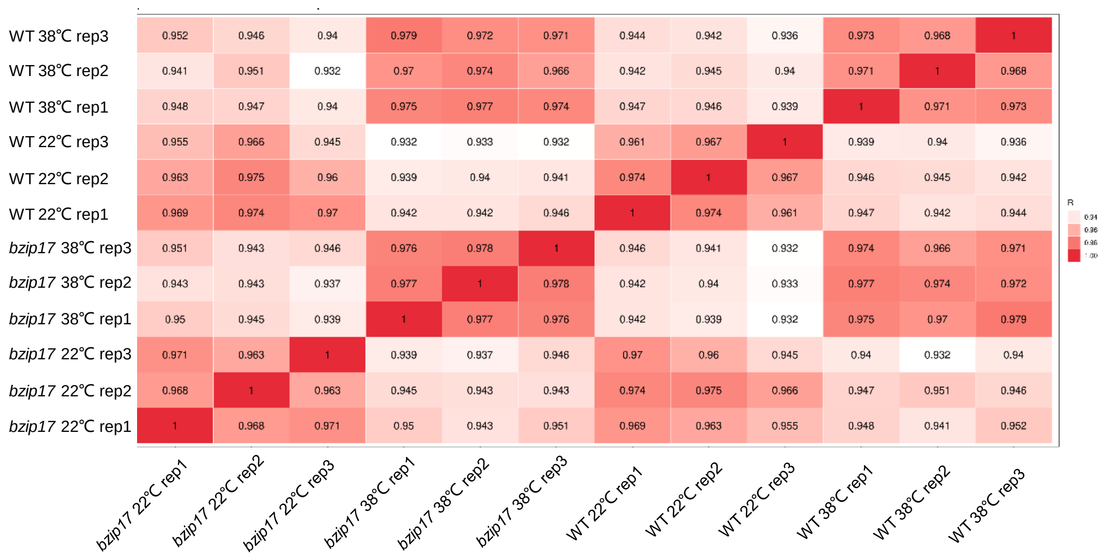

Supplemental Fig. 1. Cluster analysis of gene expression in all the tested samples in RNA-Seq analysis.

The wild-type plants (WT) and AtbZIP17 mutant (bzip17) plants were treated (38° C) or non-treated (22° C) for 6 h and subjected to RNA-Seq analysis. There were three biological replicates (Rep) in the experiment.

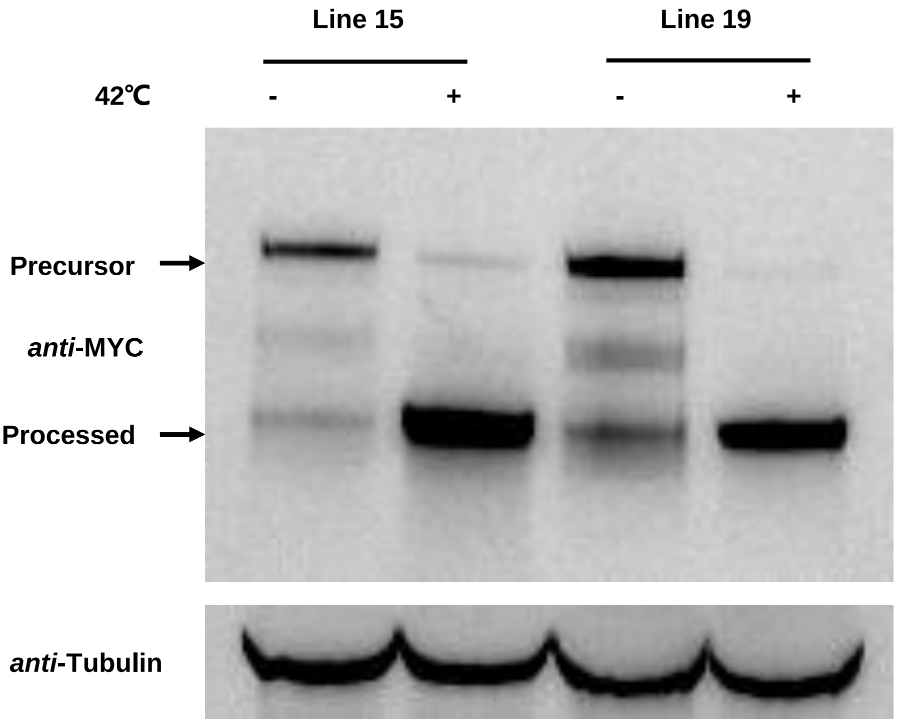

Supplemental Fig. 2. AtbZIP17 is activated under heat stress conditions.
Two transgenic lines overexpression *MYC-bZIP17* were treated or non-treated with heat stress for 2 h and total proteins were extracted for detecting protein processing using anti-MYC antibody in western blots. The precursor and processed forms are indicated with arrows.

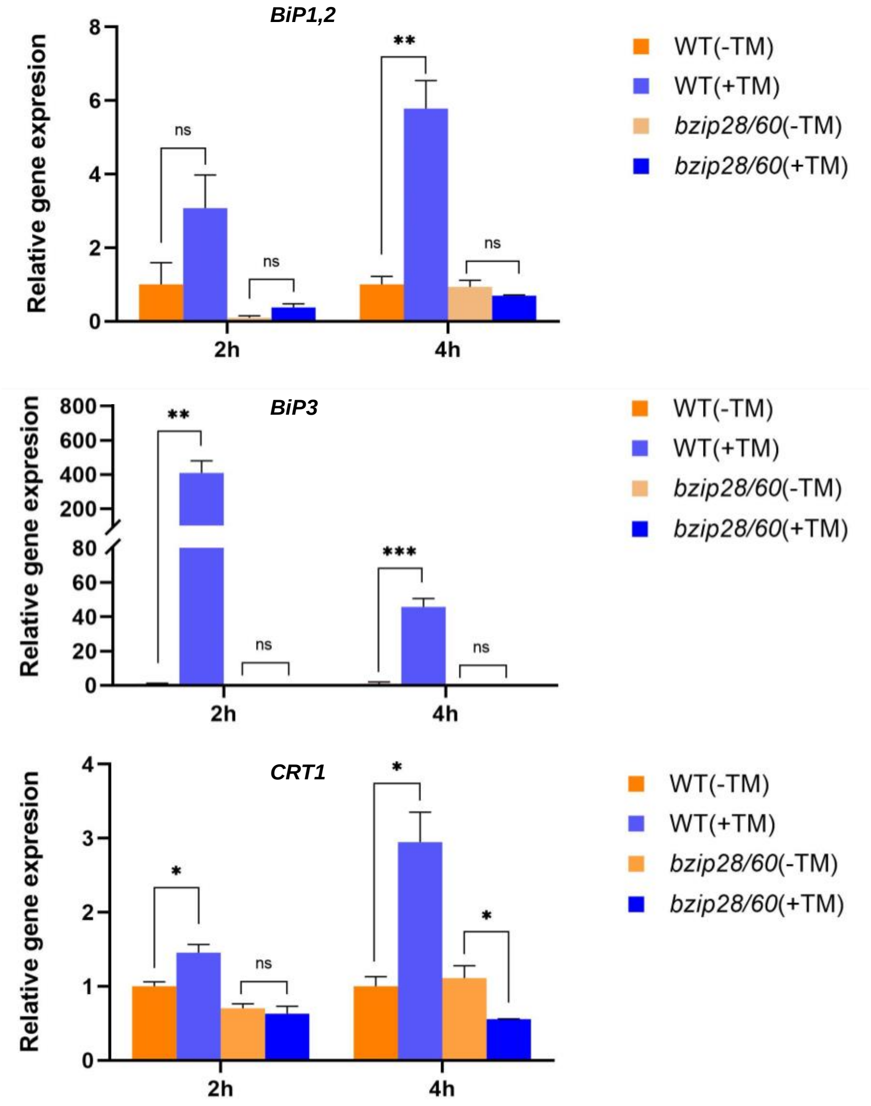

Supplemental Fig. 3. Mutations of both *AtbZIP28* and *AtbZIP60* block the up-regulation of UPR marker genes.

Wildtype (WT) and *bzip28 bzip60* double mutant plants were treated or non-treated with ER stress inducer tunicamycin (TM) for 2 h and 4 h, respectively, and total RNAs were extracted for quantitative RT-PCR (RT-qPCR) analysis of UPR marker genes. Relative gene expression is the expression level of each gene normalized to that in the WT plants, both of which were normalized to that of *ACTIN*. The bars depict the *SE* (n=3). ***, ($P < 0.001$); **, ($P < 0.01$); *, ($P < 0.05$); ns, (not significant at $P < 0.05$) in T-tests.
